# Admission respiratory status predicts mortality in COVID‐19

**DOI:** 10.1111/irv.12869

**Published:** 2021-05-24

**Authors:** Neal A. Chatterjee, Paul N. Jensen, Andrew W. Harris, Daniel D. Nguyen, Henry D. Huang, Richard K. Cheng, Jainy J. Savla, Timothy R. Larsen, Joanne Michelle D. Gomez, Jeanne M. Du‐Fay‐de‐Lavallaz, Rozenn N. Lemaitre, Barbara McKnight, Sina A. Gharib, Nona Sotoodehnia

**Affiliations:** ^1^ Division of Cardiology University of Washington Seattle WA USA; ^2^ Cardiovascular Health Research Unit University of Washington Seattle WA USA; ^3^ Division of Cardiology Rush University Medical Center Chicago IL USA; ^4^ Department of Biostatistics University of Washington Seattle WA USA; ^5^ Division of Pulmonary, Critical Care, and Sleep Medicine University of Washington Seattle WA USA

**Keywords:** COVID‐19, death, epidemiology, hypoxemia, respiratory rate

## Abstract

COVID‐19 has significant case fatality. Glucocorticoids are the only treatment shown to improve survival, but only among patients requiring supplemental oxygen. WHO advises patients to seek medical care for “trouble breathing,” but hypoxemic patients frequently have no respiratory symptoms. Our cohort study of hospitalized COVID‐19 patients shows that respiratory symptoms are uncommon and not associated with mortality. By contrast, objective signs of respiratory compromise—oxygen saturation and respiratory rate—are associated with markedly elevated mortality. Our findings support expanding guidelines to include at‐home assessment of oxygen saturation and respiratory rate in order to expedite life‐saving treatments patients to high‐risk COVID‐19 patients.

## 1 SHORT REPORT

COVID‐19 is a global pandemic with significant case fatality, often due to respiratory failure. Few treatments have improved morbidity,^1^, ^2^ and the only pharmacologic intervention reported to improve survival is use of glucocorticoids in hospitalized COVID‐19 patients requiring supplemental oxygen.^3^ The National Institutes of Health recommends maintaining oxygen saturation levels at 92%‐96% for COVID‐19 patients.^4^ COVID‐19 patients often have unrecognized hypoxemia without experiencing overt respiratory symptoms^5^, ^6^ resulting in a missed opportunity to institute early, potentially life‐saving treatment. While the Centers for Disease Control (CDC) and World Health Organization (WHO) advise COVID‐19 patients to seek medical attention if they have “trouble breathing,” there are no current guidelines to assess objective signs of respiratory insufficiency at home, such as oxygen saturation or respiratory rate.^7^ We evaluated whether initial oxygen saturation and respiratory rate are associated with all‐cause mortality in hospitalized COVID‐19 patients.

Study cohort included consecutive COVID‐19 patients ≥18 years of age hospitalized at University of Washington, Seattle, WA, or Rush University, Chicago, IL medical centers between March 1, and June 8, 2020. Exclusion criteria included persons choosing “comfort measures only” at hospital admission and those without clinical symptoms of COVID‐19 admitted for non‐COVID‐19‐related medical issues. SARS‐CoV‐2 infection was confirmed using qRT‐PCR. Study was approved by Institutional Review Boards at both institutions.

Patient information and clinical outcomes were collected by physician chart review. Primary outcome was all‐cause in‐hospital mortality. Poisson regression models with robust standard errors were used to calculate relative risks (RR) and 95% confidence intervals (CI) for the associations of oxygen saturation and respiratory rate with the mortality. Models were adjusted for age, sex, race, health system, nursing home residence, smoking status, hypertension, diabetes, body mass index [BMI], pulmonary disease, and cardiovascular disease. In secondary analyses, linear regression models were used to examine associations of candidate risk factors with admission oxygen saturation and respiratory rate. Multiple imputation with chained equations was used to impute missing BMI values (n = 46).

During the study, 1,095 individuals were hospitalized with symptomatic COVID‐19. Patients had mean age of 58, were mostly men (62%), and commonly had comorbidities (Table 1). While patients frequently had hypoxemia (mean oxygen saturation of 91%) and tachypnea (mean respiratory rate of 23 breaths per minute) on presentation, few reported shortness of breath (10%) or cough (25%) regardless of oxygen saturation. The most common symptom at presentation was fever (73%). Patient respiratory symptoms and symptomatic fever were not associated with mortality.

**TABLE 1:** Baseline characteristics

	Total (N = 1095)
Age, years	58 ± 17
Male sex, n (%)	684 (62%)
Race, n (%)	
White	235 (21%)
Hispanic	410 (37%)
Black	384 (35%)
Asian	51 (5%)
Unknown	15 (1%)
Nursing Home Resident, n (%)	109 (10%)
Body mass index,^a^ kg/m^2^	32 ± 9
Prior Medical History, n (%)	
Hypertension	588 (54%)
Diabetes mellitus	357 (33%)
Coronary disease	129 (12%)
Heart Failure	126 (12%)
Myocardial infarction	35 (3%)
Peripheral arterial disease	33 (3%)
Stroke	98 (9%)
Chronic Kidney disease	187 (17%)
Chronic Liver failure	43 (4%)
Admission Characteristics	
Oxygen saturation, %^b^	91 ± 9
Supplemental oxygen use, n (%)	819 (75%)
Heart rate, beats per minute	93 ± 19
Respiratory rate, breaths per minute	23 ± 6
Temperature, F	99.7 ± 2
Systolic blood pressure, mmHg	128 ± 22
Diastolic blood pressure, mmHg	75 ± 15
Symptoms at Presentation	
Fever	792 (73%)
Shortness of breath	112 (10%)
Cough	282 (26%)
Myalgia	237 (22%)
Fatigue	275 (25%)
GI symptoms	527 (48%)
Chest pain	118 (11%)
Syncope	20 (2%)
Days symptomatic prior to admission	6 ± 4

Note

Continuous variables are reported as mean ±standard deviation.

^a^ Body mass index information was missing for 46 patients; supplemental oxygen use was missing for 3 patients; temperature was missing for 3 patients; systolic and diastolic blood pressure was missing for 1 patient; symptoms at presentation were missing for 6 patients; and days symptomatic prior to admission was missing for 13 patients. Multiple imputation with chained equations was used to impute missing values of BMI (n = 46) using information on admission oxygen saturation, respiratory rate, age, sex, race, health system, and prevalent hypertension, diabetes, and cardiovascular disease, and in‐hospital mortality.

^b^ Oxygen saturation reflects measurement upon patient presentation prior to initiation of supplemental oxygen.

**FIGURE 1:**
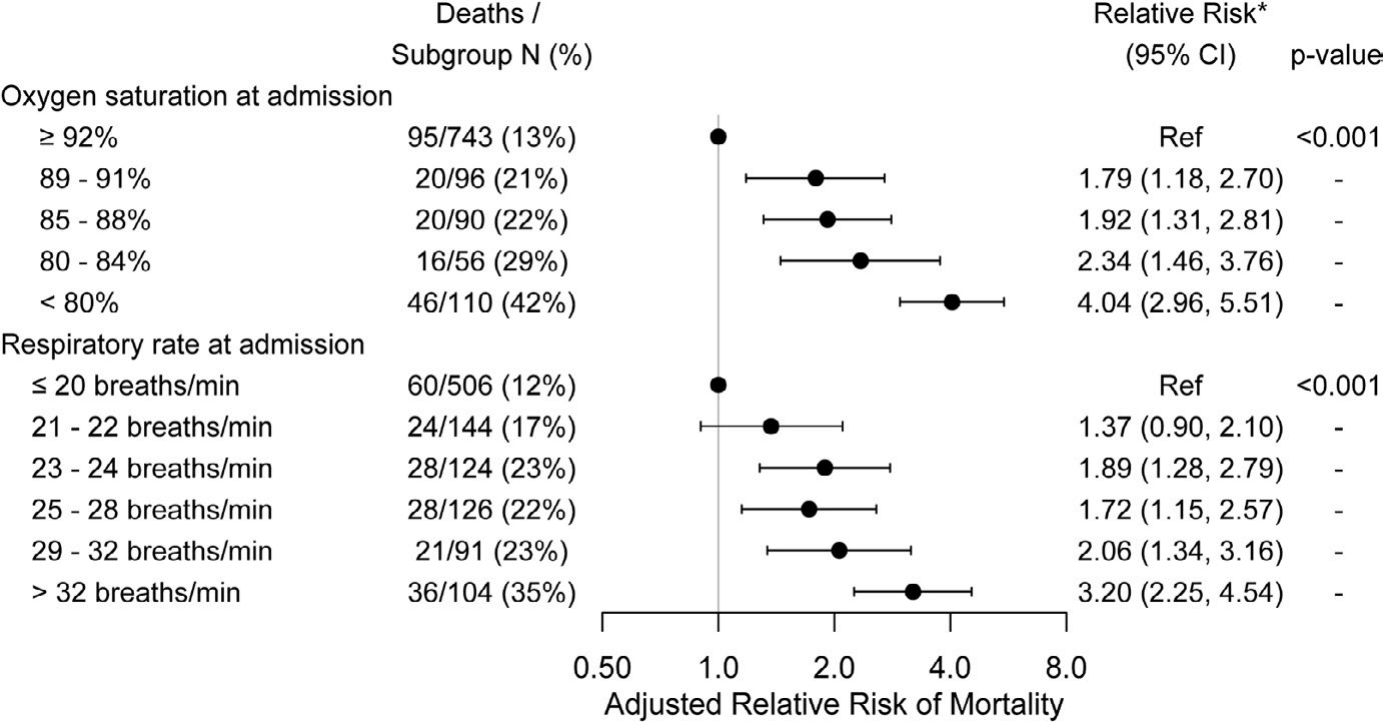
Association of oxygen saturation and respiratory rate on admission with in‐hospital mortality. *Multivariable adjustment is for age, sex, race, health system, hypertension, diabetes mellitus, body mass index, pulmonary disease, cardiovascular disease, smoking, and nursing home residence

Overall, 197 patients died in hospital. After adjustment for risk factors, both hypoxemia and tachypnea were associated with mortality risk (Figure 1). Compared to normoxemic patients, those who were hypoxemic (oxygen saturation <92%) had a 1.8‐ to 4.0‐fold increased mortality risk, depending on initial oxygen saturation. Similarly, compared to patients with normal respiratory rate (≤20 beats per minute), those with respiratory rates >22 breaths per minute were at 1.9‐ to 3.2‐fold elevated mortality risk. Nearly, all hypoxemic (99%) and tachypneic (98%) patients required supplemental oxygen administration during hospitalization. By contrast, other clinical signs at presentation, including temperature, heart rate, and blood pressure, were not associated with mortality. Findings were similar among subgroups stratified by demographic and clinical characteristics.

No clinical or demographic characteristic was associated with admission oxygen saturation or respiratory rate with one notable exception. BMI was associated with both lower oxygen saturation (β‐Coefficient −0.95, [CI: −1.32, −0.57] per 5 kg/m^2^ higher BMI, *P* < 0.001) and more rapid respiratory rate (β‐Coefficient 0.36 [CI:0.14, 0.59] per 5 kg/m^2^ higher BMI, *P* = 0.001). Low admission oxygen saturation partly accounted for the higher mortality rate seen with obesity; the association between BMI and all‐cause mortality (RR 1.08 [95% CI: 1.00, 1.16] per 5 kg/m^2^ higher BMI, *P* = 0.04) was attenuated after adjustment for admission oxygen saturation (RR 1.03 [95% CI: 0.96, 1.11], *P* = 0.42).

Our study shows that indices of respiratory compromise at initial presentation that are readily measurable at home—oxygen saturation <92% or a respiratory rate >22 breaths per minute—were each associated with elevated mortality in hospitalized COVID‐19 patients. Current CDC and WHO COVID‐19 guidelines do not include recommendations for measuring oxygen saturation or respiratory rate at home; instead, they recommend patients seek medical attention if they have “trouble breathing.”^7^ In our study, only 10% of hospitalized patients reported shortness of breath, and admission respiratory symptoms were not associated with hypoxemia or mortality, underscoring that respiratory symptoms are uncommon and by themselves may not accurately identify at‐risk patients.^5^

Hypoxemia has significant therapeutic implications; only COVID‐19 patients who require supplemental oxygen benefit from medications that decrease mortality. We and others have shown that hypoxemia is often asymptomatic which may result in a delay in life‐saving treatment. Together, with our findings showing markedly increased mortality risk with hypoxemia, these data support expanding public health messaging to include at‐home assessment of oxygen saturation for COVID‐19 patients. In resource‐limited settings where oxygen saturation monitoring devices are not accessible, respiratory rate of >22 may serve as a surrogate marker for respiratory compromise. Consideration of inclusion of hypoxemia and tachypnea in guidelines as indication for COVID‐19 patients to seek medical consultation is warranted.

While the majority of patients in our study had one or more comorbidities, the increased mortality risk associated with hypoxemia and tachypnea was present irrespective of comorbidities.^6^ Obese patients have an elevated risk of silent hypoxemia and tachypnea that partly account for their elevated mortality risk and are likely to benefit most from assessing their respiratory status during acute COVID‐19.^8^

Study limitations include analysis restricted to hospitalized patients with comorbidities which may not generalize to non‐hospitalized patients. Since patient symptoms were retrospectively abstracted from medical records, symptom prevalence may be underestimated. We cannot exclude the possibility of residual confounding. Although pulse oximetry may underestimate the severity of hypoxemia in patients with darker skin tones,^9^ our study findings were robust among those of African and Hispanic ancestry. While the accuracy of at‐home pulse oximeters may be compromised at oxygen saturations less than 90%,^10^ ascertainment at the risk threshold identified in this study (92%) is attainable.

In summary, indices of respiratory compromise at initial presentation, that are readily measurable at home, are associated with markedly elevated risk of in‐hospital mortality in COVID‐19 patients. Public health strategies emphasizing the importance of at‐home surveillance of oxygen saturation and respiratory rate may identify at‐risk patients earlier and enable timely institution of life‐saving medical therapy.

## DISCLOSURES

The authors each report no disclosures relevant to the content of the manuscript.

## AUTHOR CONTRIBUTIONS

**Neal Chatterjee:** Conceptualization (equal); Formal analysis (lead); Investigation (equal); Methodology (equal); Project administration (equal); Writing‐original draft (lead); Writing‐review & editing (equal). **Paul Jensen:** Data curation (equal); Formal analysis (lead); Investigation (equal); Methodology (equal); Writing‐original draft (equal). **Andrew Harris:** Data curation (equal); Resources (equal). **Daniel D. Nguyen:** Data curation (equal); Resources (equal). **Henry Huang:** Data curation (equal); Writing‐review & editing (equal). **Richard Cheng:** Data curation (equal); Writing‐review & editing (equal). **Jainy Savla:** Data curation (equal). **Timothy Larsen:** Data curation (equal). **Joanne Michelle Gomez:** Data curation (equal); Writing‐review & editing (equal). **Jeanne Du‐Fay‐de‐Lavallaz:** Data curation (equal); Writing‐review & editing (equal). **Rozenn Lemaitre:** Investigation (supporting); Writing‐review & editing (equal). **Barbara McKnight:** Investigation (supporting); Methodology (supporting); Writing‐original draft (supporting). **Sina A Gharib:** Investigation (supporting); Supervision (supporting); Writing‐review & editing (equal). **Nona Sotoodehnia:** Conceptualization (lead); Formal analysis (equal); Investigation (equal); Project administration (lead); Supervision (lead); Writing‐original draft (equal); Writing‐review & editing (equal).

### PEER REVIEW

The peer review history for this article is available at https://publons.com/publon/10.1111/irv.12869.

## Data Availability

The data that support the findings of this study are available from the corresponding author upon reasonable request.
